# Safety and effectiveness of reduced-port laparoscopic sleeve gastrectomy in Asian morbidly obese patients

**DOI:** 10.1038/s41598-021-02999-1

**Published:** 2021-12-06

**Authors:** Yeshong Park, Young Suk Park, Sangjun Lee, So Hyun Kang, Eunju Lee, Sang-Hoon Ahn, Yun-Suhk Suh, Do Joong Park, Hyung-Ho Kim

**Affiliations:** 1 Department of Surgery, Seoul National University Bundang Hospital, 82, Gumi-ro 173beon-gil, Bundang-gu, Seongnam-si, Gyeonggi-do Republic of Korea 13620; 2 Department of Surgery, Seoul National University College of Medicine, Seoul, Korea; 3 Department of Surgery, Seoul National University Hospital, Seoul, Korea

**Keywords:** Weight management, Obesity, Outcomes research

## Abstract

Laparoscopic sleeve gastrectomy is the most frequently performed surgical intervention in patients with morbid obesity. Single-port sleeve gastrectomy (SPSG) and reduced-port sleeve gastrectomy (RPSG) are increasingly reported in the literature. This study compared the short-term outcomes of SPSG, RPSG, and conventional laparoscopic sleeve gastrectomy (CLSG). This is a single-center retrospective study of 238 morbidly obese patients, of whom 148 (62.2%) patients completed follow-up one year after surgery. Propensity score matching was performed on factors influencing the choice of approach, and fifty patients from the SPSG + RPSG and CLSG groups were successfully matched. The groups were comparable in postoperative weight loss, morbidity, pain, and resolution of obesity-related comorbidities. The percentage of excess weight loss after one year was 90.0% in the SPSG + RPSG group and 75.2% in the CLSG group (P < 0.001). Complication rates showed no significant difference. The CLSG group was superior in dyslipidemia remission (17 [37.0%] vs. 28 [63.6%], P = 0.018) in the total cohort; however, this difference disappeared after matching. Our results suggest that single-port and reduced-port approaches could be alternative choices for selected patients. As our study was limited by its retrospective nature and potential selection bias, further studies are necessary to set standardized guidelines for SPSG.

## Introduction

Obesity is a growing global health issue, and obesity-related diseases have recently gained increasing attention in Asian countries^1^. In 2019, the Korean Ministry of Health and Welfare announced that the National Health Insurance would reimburse bariatric surgery costs, reflecting the importance of surgical intervention in patients with severe obesity. Bariatric surgery for morbid obesity is associated with significant weight loss and decreased mortality, and laparoscopic sleeve gastrectomy is the most frequently performed surgical procedure worldwide^2^.

Single-incision laparoscopic surgery (SILS) was first introduced in the 1990s, and its application has been extended to various surgical procedures^3–5^. SILS has been associated with less postoperative pain, lower risk of wound infection, shorter hospital stay, and better cosmesis^6^. Laparoscopic sleeve gastrectomy is an excellent candidate for the single-incision approach, as the surgical field is confined to the left upper abdominal quadrant and the single incision wound is similar in size to the port site extension in conventional laparoscopic sleeve gastrectomy (CLSG)^7^.

Single-port sleeve gastrectomy (SPSG) and reduced port sleeve gastrectomy (RPSG) that utilizes one additional port have been increasingly reported in the literature^8,9^. However, there is still an ongoing debate on whether the technical difficulties of the single-port approach might lead to an increased risk of postoperative morbidity and suboptimal sleeve construction^7^. SILS has been implemented in various gastrectomy procedures, and favorable results were reported on single-port laparoscopic distal gastrectomy, total gastrectomy, and resectional Roux-en-Y gastric bypass^10–12^. In the present study, we compared the short-term outcomes of SPSG and RPSG *versus* CLSG in postoperative weight loss, morbidity rate, pain, and resolution of obesity-related diseases.

## Materials and methods

### Patients

**Figure 1:**
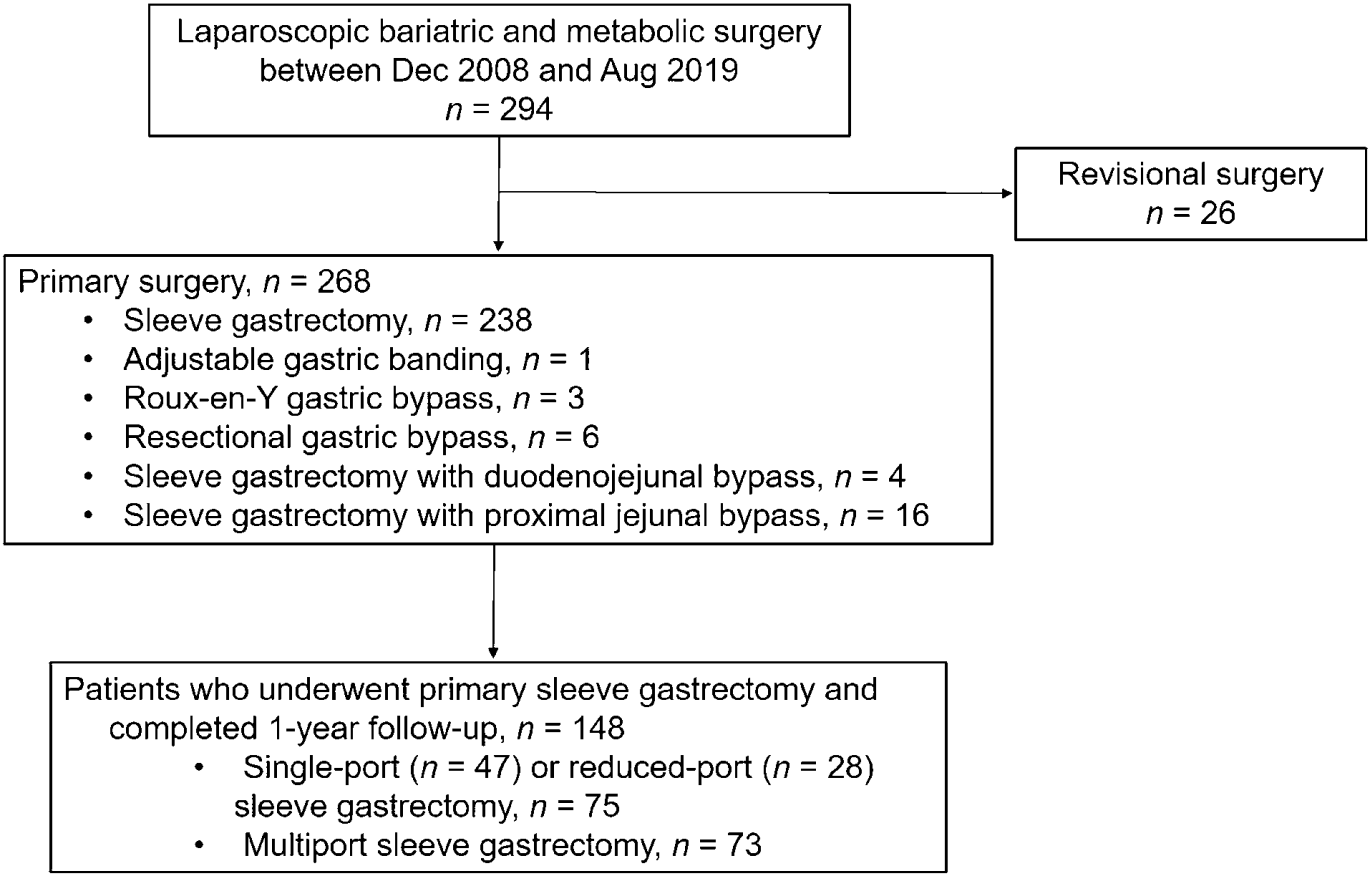
Patient selection flow diagram.

We conducted a retrospective analysis of a prospective cohort who underwent laparoscopic sleeve gastrectomy between December 2008 and August 2019 at Seoul National University Bundang Hospital. Among 238 patients included in analysis, 148 (62.2%) patients completed follow-up one year after surgery. The patient selection flow diagram is shown in Fig. 1. The indications for bariatric surgery were a body mass index (BMI) of ≥ 35 kg/m^2^ or a BMI of ≥ 30 kg/m^2^ with obesity-related comorbidities, including hypertension, type 2 diabetes mellitus, dyslipidemia, gastroesophageal reflux disease (GERD), and fatty liver disease. Patients were excluded if they had previously undergone bariatric surgery. Preoperative assessment included basic evaluation of medical history, anthropometric measurements, laboratory testing, low-dose non-enhanced abdominal computed tomography (CT), and esophagogastroduodenoscopy (EGD). CT scans were performed for preoperative evaluation of stomach anatomy and any intra-abdominal abnormalities, including the presence of hiatal hernia. EGD was performed for gastric cancer screening and H. pylori infection testing, considering the high prevalence of gastric cancer in Korea^13^. The presence of reflux esophagitis was also evaluated.

Indications for SPSG or RPSG were as follows, yet not absolute: (i) female sex, (ii) BMI ≤ 42 kg/m^2^, and (iii) no history of upper abdominal surgery except for laparoscopic cholecystectomy. Laparoscopic cholecystectomy was excluded as it generally results in adhesions only around the gallbladder bed, which do not interfere with the left upper abdominal quadrant surgical field of sleeve gastrectomy. Male patients or patients with BMI > 42 kg/m^2^ also underwent SPSG or RPSG at the patient’s request. The propensity score analysis included 47 patients who underwent SPSG, 28 who underwent RPSG, and 73 who underwent CLSG. SPSG and RPSG patients were considered as a single group as they were selected using the same inclusion criteria. All procedures performed in this study were in accordance with the ethical standards of the Institutional Review Board (IRB) of Seoul National University Bundang Hospital (IRB No. B-2105-683-102). Only anonymous patient data were collected, and informed consent for this retrospective analysis was waived by the IRB.

### Operative technique

**Figure 2:**
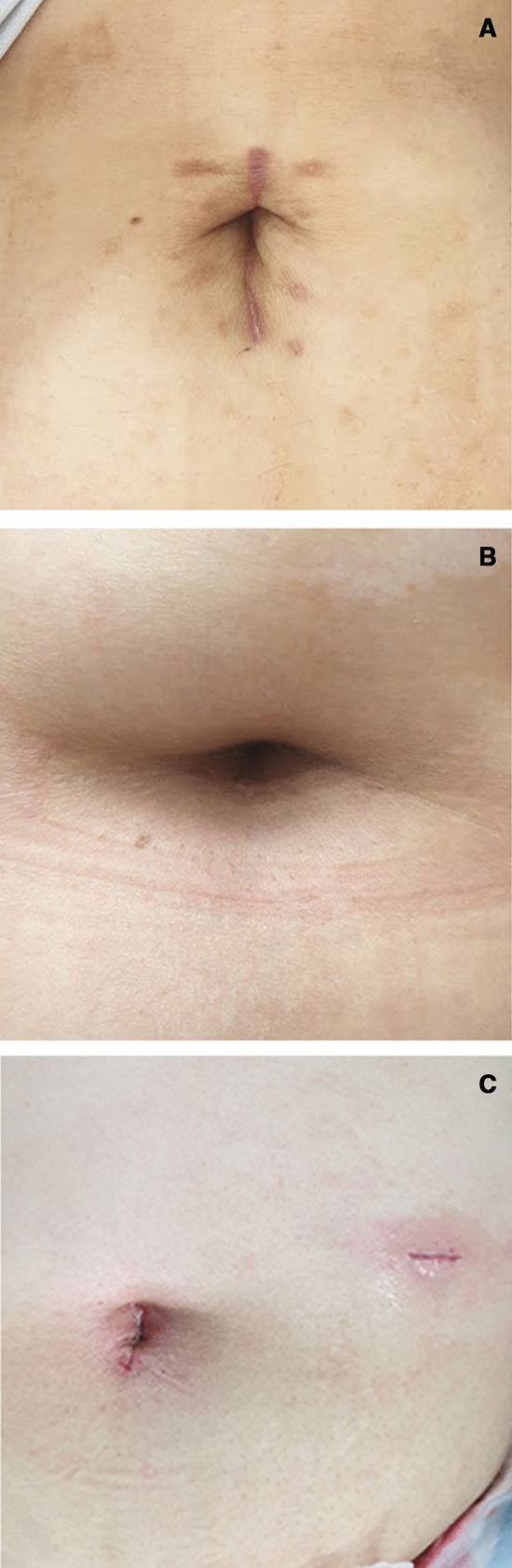
Postoperative wound after laparoscopic single-port and reduced-port sleeve gastrectomy. (**A**) Postoperative wound after laparoscopic single-port sleeve gastrectomy (2008–2018). (**B**) Postoperative wound after laparoscopic single-port sleeve gastrectomy, limited to the transumbilical level (2018–present). (**C**) Postoperative wound after laparoscopic reduced-port sleeve gastrectomy, with the main wound limited to the transumbilical level and an additional port utilized.

Early cases of sleeve gastrectomy performed at our institution were exclusively multi-port. In 2015, the single-incision approach was first implemented; from 2015 to 2019, SPSG was predominantly performed at our institution for patients meeting the inclusion criteria. However, pure single-port surgery had critical limitations due to increased interference between instruments that impeded camera view during the stapling process. To overcome this limitation, we first tried to increase the incision size; however, this resulted in poorer cosmesis and decreased patient satisfaction (Fig. 2A). Therefore, since 2019, we have limited the incision to the transumbilical level (Fig. 2B) and have utilized an additional 5- or 10-mm camera port when necessary (Fig. 2C).

**Figure 3:**
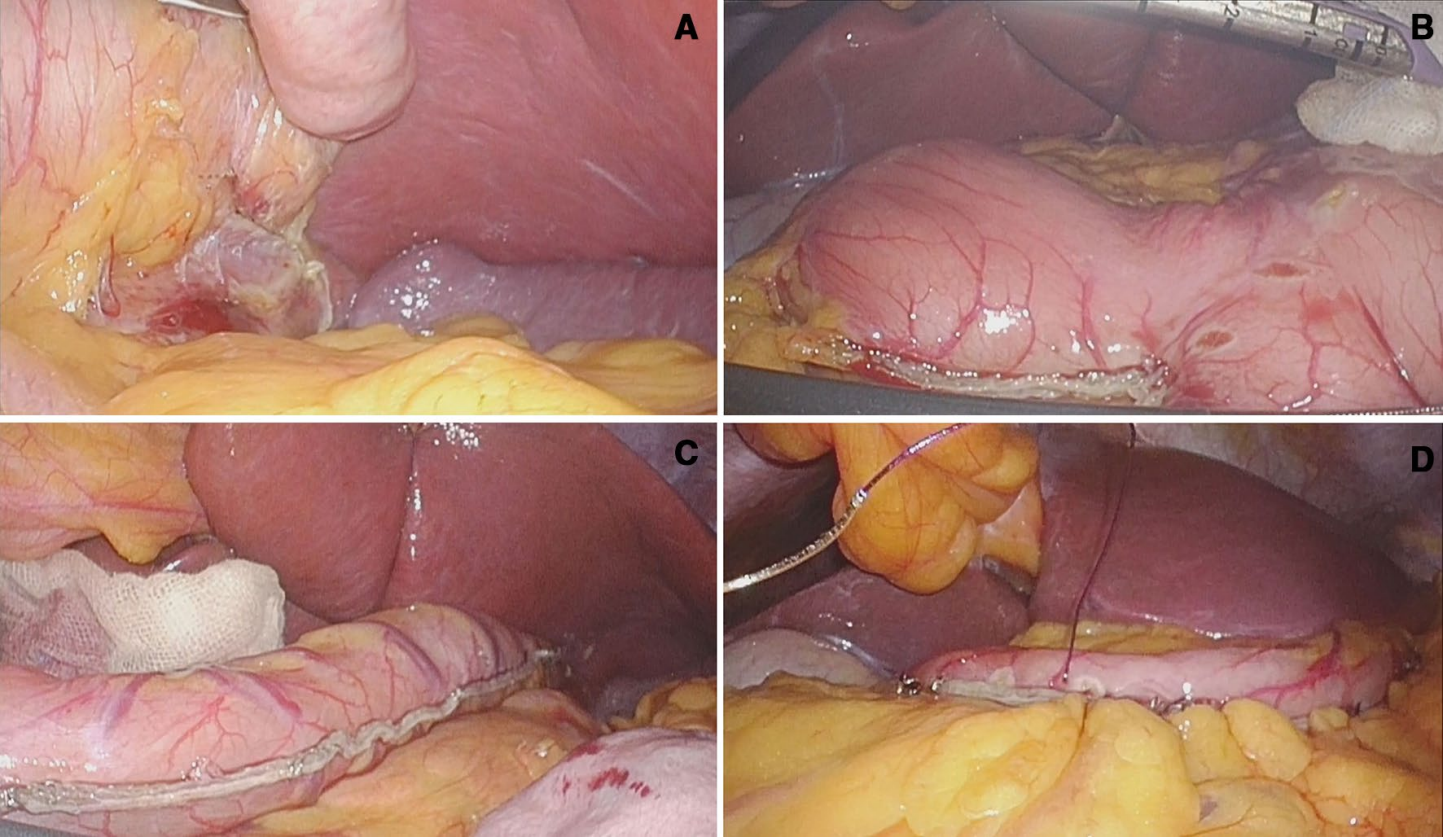
The laparoscopic reduced-port sleeve gastrectomy procedure. (**A**) Fundus dissection. (**B**) 1st stapling with an endoscopic stapler. (**C**) Completion of gastrectomy calibrated with a 36-Fr orogastric suction bougie. (**D**) Final view after remnant stomach was fixed to the greater omentum.

All surgical procedures were identical in the CLSG, RPSG, and SPSG groups, except for trocar insertion. All patients were placed in the lithotomy position under general anesthesia. A 5- or 10-mm flexible tip laparoscope and a thermofusion device were used. After calibration with a 36-Fr orogastric suction bougie, the stomach was transected using an endoscopic stapler. After sleeve construction, the remnant stomach was fixed to the greater omentum using continuous sutures to prevent axial twisting and postoperative sleeve stenosis (Fig. 3). We usually did not place abdominal drains after surgery. In all cases, fascia closure was routinely performed for incisions larger than 10 mm.

In SPSG, all procedures were performed via a multi-channel single-access device introduced through a 2–4 cm transumbilical incision. RPSG was performed utilizing one additional trocar to the SPSG procedure. The additional trocar was placed in the left middle quadrant and was primarily used for insertion of the laparoscope camera. CLSG was performed using one 5-mm and three 12-mm trocars.

### Definition of comorbidities

Hypertension was defined as systolic blood pressure ≥ 140 mmHg, diastolic blood pressure ≥ 90 mmHg, or current use of antihypertensive medication. Diabetes mellitus (DM) was defined as fasting blood sugar ≥ 7.0 mmol/L, hemoglobin A1c (HbA1c) ≥ 6.5%, or current administration of oral hypoglycemic agents or subcutaneous insulin. Dyslipidemia was defined as low-density lipoprotein cholesterol (LDL-C) ≥ 8.9 mmol/L, high-density lipoprotein-cholesterol (HDL-C) < 2.2 mmol/L, triglyceride (TG) ≥ 11.1 mmol/L, or current use of lipid-lowering medication. Psychological disorders included mood disorders, anxiety disorders, schizophrenia, insomnia, and eating disorders. GERD was defined as follows: reflux symptoms necessitating daily proton pump inhibitor (PPI) intake and/or esophagitis on endoscopic evaluation.

### Data collection

Demographic data were obtained from an electronic database of the medical records. This included the clinical characteristics of the patients and follow-up data on weight loss, postoperative complications, and resolution of obesity-related comorbidities. Follow-up visits were scheduled 1, 3, 6, and 12 months after the operation.

### Measurement of postoperative outcomes

Weight loss outcomes were reported using the percent of total weight loss (%TWL = [(initial weight) – (postoperative weight)]/([(initial weight)] × 100) and percent excess weight loss (%EWL = [(initial weight) – (postoperative weight)]/[(initial weight) – (ideal weight]). %EWL was based on a patient ideal weight that results in a BMI of 25 kg/m^2^.

Postoperative complications and resolution of comorbidities were reported following the American Society for Metabolic and Bariatric Surgery (ASMBS) outcome reporting standards^14^. Early complications were defined as complications that occurred within the first 30 days after surgery, and late complications were defined as complications that occurred after over 30 days. Readmission and reoperation events were also recorded.

Postoperative pain was evaluated using the numeric rating score (NRS), with 0 indicating no pain and 10 indicating the worst possible pain. Patients were asked to report on the level of pain several times per day, and the highest reported score was used for analysis.

### Statistical analysis

Propensity score matching was performed to adjust for differences in demographic and anthropometric characteristics between the two groups and reduce the effect of selection bias. Matching factors included sex, height, weight, and BMI. All matching factors were well balanced after propensity score matching (Supplementary Fig. S1). Continuous variables were compared by Student’s *t*-test before matching and paired-samples *t*-test after matching; categorical variables were compared by the chi-squared test or Fisher’s exact test. Statistical analysis were performed using IBM SPSS Statistics for Windows, Version 23.0 (IBM Corp., Armonk, NY, USA). Differences were considered statistically significant at *P* < 0.05.

### Ethical approval

All procedures performed in studies involving human participants were in accordance with the ethical standards of the institutional and/or national research committee and with the 1964 Helsinki declaration and its later amendments or comparable ethical standards.

## Results

### Patient characteristics

The clinical characteristics of the study participants are summarized in Table 1. As the inclusion criteria for SPSG and RPSG included sex and BMI, the two groups differed significantly in sex, height, weight, and BMI but were similar in the incidence of comorbidities. The CLSG group included more males (14 [18.7%] vs. 38 [52.1%], *P* < 0.001), taller patients (163.4 ± 8.0 vs. 168.2 ± 10.0 cm, *P* = 0.002), and patients with higher weight (101.2 ± 19.6 vs. 115.9 ± 20.1 kg, *P* < 0.001) and BMI (37.7 ± 5.1 vs. 40.9 ± 5.8 kg/m^2^, *P* = 0.001). Fifty patients in each group were successfully matched by propensity score adjustment. Analysis of the SPSG and RPSG subgroups before propensity score adjustment found no difference in baseline characteristics (Supplementary Table S1).

**Table 1:** Clinical characteristics of the total and propensity score-matched cohorts.

	Total cohort				Matched cohort			
	SPSG + RPSG (*n* = 75)	CLSG (*n* = 73)	Total (*n* = 148)	*P*-value	SPSG + RPSG (*n* = 50)	CLSG (*n* = 50)	Total (*n* = 100)	*P*-value
**Sex [*n*, (%)]**				< *0.001*				0.288
Female	61 (81.3)	35 (47.9)	96 ± 64.9		36 ± 72.0	31 ± 62.0	67 ± 67.0	
Male	14 (18.7)	38 (52.1)	52 ± 35.1		14 ± 28.0	19 ± 38.0	33 ± 33.0	
**Age [years, mean ± SD]**	38.0 ± 11.4	38.3 ± 11.5	38.2 ± 11.4	0.850	35.7 ± 10.6	39.4 ± 11.3	37.6 ± 11.0	0.096
**Height [cm, mean ± SD]**	163.4 ± 8.0	168.2 ± 10.0	165.8 ± 9.3	*0.002*	164.9 ± 8.5	167.0 ± 10.4	166.0 ± 9.5	0.282
**Weight [kg, mean ± SD]**	101.2 ± 19.6	115.9 ± 20.1	108.4 ± 21.1	< *0.001*	107.4 ± 20.7	108.4 ± 17.8	107.9 ± 19.2	0.799
**BMI [kg/m**^**2**^**, mean ± SD]**	37.7 ± 5.1	40.9 ± 5.8	39.3 ± 5.7	*0.001*	39.3 ± 5.5	38.8 ± 4.4	39.0 ± 5.0	0.582
**Comorbidities [*n*, (%)]**								
Hypertension				0.460				0.164
No	35 (46.7)	27 (37.0)	62 (41.9)		26 (52.0)	18 (36.0)	44 (44.0)	
Diagnosed	30 (40.0)	36 (49.3)	66 (44.6)		22 (44.0)	26 (52.0)	48 (48.0)	
Incidentally found	10 (13.3)	10 (13.7)	20 (13.5)		2 (4.0)	6 (12.0)	8 (8.0)	
Diabetes				0.510				0.255
No	53 (70.7)	45 (61.6)	98 (66.2)		38 (76.0)	30 (60.0)	68 (68.0)	
Diagnosed	18 (24.0)	23 (31.5)	41 (27.7)		10 (20.0)	16 (32.0)	26 (26.0)	
Incidentally found	4 (5.3)	5 (6.8)	9 (6.1)		2 (4.0)	4 (8.0)	6 (6.0)	
Dyslipidemia				0.339				0.170
No	29 (38.7)	29 (39.7)	58 (39.2)		22 (44.0)	22 (44.0)	44 (44.0)	
Diagnosed	18 (24.0)	24 (32.9)	42 (28.4)		9 (18.0)	16 (32.0)	25 (25.0)	
Incidentally found	28 (37.3)	20 (27.4)	48 (32.4)		19 (38.0)	12 (24.0)	31 (31.0)	
NAFLD	32 (42.7)	37 (50.7)	69 (46.6)	0.328	22 (44.0)	23 (46.0)	45 (45.0)	0.841
Psychological disorder	19 (25.3)	14 (19.2)	33 (22.3)	0.368	14 (28.0)	11 (22.0)	25 (25.0)	0.488
**GERD**								
Symptoms only	3 (4.0)	5 (6.8)	8 (5.4)	0.491	2 (4.0)	5 (10.0)	7 (7.0)	0.436
Esophagitis on endoscopy	14 (18.9)	21 (28.8)	35 (23.8)	0.161	13 (26.0)	13 (26.0)	26 (26.0)	0.999
OSA	15 (20.0)	22 (30.1)	37 (25.0)	0.154	12 (24.0)	13 (26.0)	25 (25.0)	0.817
Lung disease	2 (2.7)	2 (2.7)	4 (2.7)	0.999	1 (2.0)	1 (2.0)	2 (2.0)	0.999

Significant values are italics.

*SPSG* single-port sleeve gastrectomy, *RPSG* reduced port sleeve gastrectomy, *CLSG* conventional laparoscopic sleeve gastrectomy, *SD* standard deviation, *BMI* body mass index, *NAFLD* non-alcoholic fatty liver disease, *GERD* gastroesophageal reflux disease, *OSA* obstructive sleep apnea.

### Weight loss

In the total patient cohort, superior weight loss at one month (10.5 ± 3.7 vs. 12.0 ± 3.4 kg, *P* = 0.015) and three months (18.1 ± 5.4 vs. 20.8 ± 6.3 kg, *P* = 0.009) was observed in the CLSG group (Table 2). The SPSG + RPSG group showed a superior % EWL at three months (58.3 ± 21.6 vs. 50.9 ± 20.5, *P* = 0.040), six months (80.3 ± 25.3 vs. 65.9 ± 26.5, *P* = 0.001), and 12 months (90.0 ± 29.8 vs. 75.2 ± 29.9, *P* = 0.003). These differences were not observed after group matching. In the subgroup analysis, SPSG and RPSG patients showed no difference in weight loss, BMI reduction, %TWL, and %EWL before and after propensity score matching (Table 3).

**Table 2:** Weight loss in the total and propensity score-matched cohorts at 1, 3, 6, and 12-month follow-up after surgery.

	Total cohort				Matched cohorts			
	SPSG + RPSG (*n* = 75)	CLSG (*n* = 73)	Total (*n* = 148)	*P*-value	SPSG + RPSG (*n* = 50)	CLSG (*n* = 50)	Total (*n* = 100)	*P*-value
**Weight loss (kg, mean ± SD)**								
1-month	10.5 ± 3.7	12.0 ± 3.4	11.2 ± 3.6	*0.015*	11.3 ± 3.8	11.5 ± 3.4	11.4 ± 3.6	0.770
3-month	18.1 ± 5.4	20.8 ± 6.3	19.5 ± 6.0	*0.009*	19.7 ± 5.4	19.5 ± 6.4	19.6 ± 5.9	0.883
6-month	25.3 ± 8.3	27.6 ± 9.4	26.4 ± 8.9	0.142	27.3 ± 8.9	25.7 ± 8.7	26.5 ± 8.8	0.362
12-month	28.5 ± 10.8	31.4 ± 11.2	29.9 ± 11.1	0.112	30.5 ± 11.7	29.7 ± 11.3	30.1 ± 11.5	0.708
**BMI reduction (kg/m**^**2**^**, mean ± SD)**								
1-month	3.9 ± 1.3	4.2 ± 1.1	4.0 ± 1.2	0.105	4.1 ± 1.3	4.1 ± 1.1	4.1 ± 1.2	0.998
3-month	6.8 ± 1.9	7.3 ± 1.8	7.0 ± 1.9	0.114	7.2 ± 1.9	6.9 ± 1.8	7.1 ± 1.9	0.456
6-month	9.5 ± 2.8	9.7 ± 2.9	9.6 ± 2.9	0.668	10.0 ± 3.1	9.2 ± 2.8	9.6 ± 2.9	0.160
12-month	10.6 ± 3.7	11.1 ± 3.7	10.8 ± 3.7	0.500	11.2 ± 4.1	10.6 ± 3.9	10.9 ± 4.0	0.479
**%TWL (%, mean ± SD)**								
1-month	10.3 ± 3.1	10.4 ± 2.7	10.4 ± 2.9	0.769	10.5 ± 3.1	10.7 ± 2.7	10.6 ± 2.9	0.746
3-month	17.8 ± 4.0	17.9 ± 4.3	17.9 ± 4.2	0.947	18.3 ± 4.0	18.0 ± 4.5	18.1 ± 4.3	0.719
6-month	24.8 ± 6.0	23.5 ± 6.5	24.2 ± 6.2	0.218	25.2 ± 6.3	23.4 ± 6.3	24.3 ± 6.3	0.190
12-month	27.9 ± 8.2	26.9 ± 8.2	27.4 ± 8.2	0.469	28.2 ± 8.8	27.1 ± 8.6	27.6 ± 8.7	0.528
**%EWL (%, mean ± SD)**								
1-month	34.3 ± 15.9	30.2 ± 14.2	32.3 ± 15.2	0.111	32.6 ± 17.1	33.3 ± 15.4	33.0 ± 16.1	0.843
3-month	58.3 ± 21.6	50.9 ± 20.5	54.5 ± 21.3	*0.040*	55.7 ± 22.4	55.5 ± 22.2	55.6 ± 22.2	0.972
6-month	80.3 ± 25.3	65.9 ± 26.5	73.2 ± 26.8	*0.001*	75.0 ± 25.0	71.1 ± 28.3	73.0 ± 26.7	0.489
12-month	90.0 ± 29.8	75.2 ± 29.9	82.7 ± 30.6	*0.003*	84.3 ± 29.0	81.3 ± 31.8	82.8 ± 30.3	0.633

Significant values are italics.

*SPSG* single-port sleeve gastrectomy, *RPSG* reduced port sleeve gastrectomy, *CLSG* conventional laparoscopic sleeve gastrectomy, *BMI* body mass index, *%TWL* percent of total weight loss, [(initial weight) – (postoperative weight)]/([(initial weight)] × 100; %EWL, percent excess weight loss, [(initial weight) – (postoperative weight)]/[(initial weight) – (ideal weight)].

**Table 3:** Subgroup analysis of single-port and reduced-port laparoscopic sleeve gastrectomy patients for weight loss at 1, 3, 6, and 12-month follow-up after surgery.

	Single-port (*n* = 47)	Reduced port (*n* = 28)	Total (*n* = 75)	*P*-value
**Weight loss (kg, mean ± SD)**				
1-month	10.3 ± 3.9	10.9 ± 3.4	10.5 ± 3.7	0.496
3-month	18.1 ± 5.7	18.2 ± 4.9	18.1 ± 5.4	0.977
6-month	25.1 ± 8.8	25.8 ± 7.7	25.3 ± 8.3	0.725
12-month	28.3 ± 11.7	28.8 ± 9.2	28.5 ± 10.8	0.842
**BMI reduction (kg/m**^**2**^**, mean ± SD)**				
1-month	3.8 ± 1.3	4.1 ± 1.2	3.9 ± 1.3	0.259
3-month	6.7 ± 1.9	6.9 ± 1.8	6.8 ± 1.9	0.660
6-month	9.3 ± 2.9	9.7 ± 2.8	9.4 ± 2.8	0.497
12-month	10.4 ± 3.9	10.9 ± 3.4	10.6 ± 3.7	0.593
**%TWL (%, mean ± SD)**				
1-month	9.9 ± 3.2	11.0 ± 2.8	10.3 ± 3.1	0.101
3-month	17.5 ± 4.0	18.5 ± 4.1	17.8 ± 4.0	0.309
6-month	24.1 ± 5.9	26.2 ± 6.0	24.8 ± 6.0	0.167
12-month	27.1 ± 8.4	29.2 ± 7.9	27.9 ± 8.2	0.267
**%EWL (%, mean ± SD)**				
1-month	33.3 ± 18.6	35.9 ± 9.6	34.3 ± 15.9	0.430
3-month	57.5 ± 24.6	59.7 ± 15.3	58.3 ± 21.6	0.691
6-month	77.8 ± 27.4	84.7 ± 20.8	80.3 ± 25.3	0.266
12-month	87.4 ± 31.0	94.4 ± 27.5	90.0 ± 29.8	0.327

*BMI* body mass index, *%TWL* percent of total weight loss, [(initial weight) – (postoperative weight)]/([(initial weight)] × 100; *%EWL* percent excess weight loss, [(initial weight) – (postoperative weight)]/[(initial weight) – (ideal weight].

### Postoperative morbidity

Postoperative complication rates were similar in the two groups before and after matching (Table 4). There was one case of multi-port conversion in the SPSG group due to intraoperative bleeding. Readmission and reoperation rates in the two groups showed no difference before and after matching.

**Table 4:** Postoperative complications in the total and propensity score-matched cohorts.

	Total cohort				Matched cohort			
	SPSG + RPSG (*n* = 75)	CLSG (*n* = 73)	Total (*n* = 148)	*P*-value	SPSG + RPSG (*n* = 50)	CLSG (*n* = 50)	Total (*n* = 100)	*P*-value
**Major complication [*n*, (%)]**								
Early (≤ 30 days)	0 (0.0)	2 (2.7)	2 (1.4)	0.242	0 (0.0)	2 (4.0)	2 (2.0)	0.495
Late (> 30 days)	1 (1.3)	1 (1.4)	2 (1.4)	0.999	1 (2.0)	1 (2.0)	2 (2.0)	0.999
**Minor complication [*n*, (%)]**								
Early (≤ 30 days)	7 (9.3)	6 (8.2)	13 (8.8)	0.999	1 (2.0)	5 (10.0)	6 (6.0)	0.204
Late (> 30 days)	3 (4.0)	1 (1.4)	4 (2.7)	0.620	3 (6.0)	0 (0.0)	3 (3.0)	0.242
**Complication type [*n*, (%)]**								
Bleeding	0 (0.0)	2 (2.7)	2 (1.4)	0.242	0 (0.0)	2 (4.0)	2 (2.0)	0.495
Trocar site hernia	1 (1.3)	0 (0.0)	1 (0.7)	0.999	1 (2.0)	0 (0.0)	1 (1.0)	0.999
Respiratory failure	0 (0.0)	1 (1.4)	1 (0.7)	0.493	0 (0.0)	1 (2.0)	1 (1.0)	0.999
Nausea and vomiting	6 (8.0)	1 (1.4)	7 (4.7)	0.116	2 (4.0)	1 (2.0)	3 (3.0)	0.999
Stricture/obstruction	0 (0.0)	3 (4.1)	3 (2.0)	0.117	0 (0.0)	2 (4.0)	2 (2.0)	0.495
Surgical site infection	3 (4.0)	2 (2.7)	5 (3.4)	0.999	1 (2.0)	1 (2.0)	2 (2.0)	0.999
Acute renal failure	1 (1.3)	0 (0.0)	1 (0.7)	0.999	1 (2.0)	0 (0.0)	1 (1.0)	0.999
Fluid collection	0 (0.0)	1 (1.4)	1 (0.7)	0.493	0 (0.0)	1 (2.0)	1 (1.0)	0.999
**Readmission [*n*, (%)]**	3 (4.0)	4 (5.5)	7 (4.7)	0.717	2 (4.0)	2 (4.0)	4 (4.0)	0.999
**Reoperation [*n*, (%)]**	4 (5.3)	3 (4.1)	7 (4.7)	0.999	4 (8.0)	3 (6.0)	7 (7.0)	0.999

*SPSG* single-port sleeve gastrectomy, *RPSG* reduced port sleeve gastrectomy, *CLSG* conventional laparoscopic sleeve gastrectomy.

Subgroup analysis showed that patients in the SPSG group experienced a significantly higher early minor complication rate than the RPSG group (7 [14.9%] vs. 0 [0.0%], *P* = 0.041), minor nausea and vomiting in all (Table 5). Late minor complications were also exclusively found in the SPSG group, although the difference was insignificant (3 [6.4%] vs. 0 [0.0%], *P* = 0.289). The readmission and reoperation rates were similar.

**Table 5:** Subgroup analysis of single-port and reduced-port laparoscopic sleeve gastrectomy patients for postoperative complications.

	Single-port (*n* = 47)	Reduced port (*n* = 28)	Total (*n* = 75)	*P*-value
**Major complication [*n*, (%)]**				
Early (≤ 30 days)	0 (0.0)	0 (0.0)	0 (0.0)	
Late (> 30 days)	1 (2.1)	0 (0.0)	1 (1.3)	0.999
**Minor complication [*n*, (%)]**				
Early (≤ 30 days)	7 (14.9)	0 (0.0)	7 (9.3)	*0.041*
Late (> 30 days)	3 (6.4)	0 (0.0)	3 (4.0)	0.289
**Complication type [*n*, (%)]**				
Trocar site hernia	1 (2.1)	0 (0.0)	1 (1.3)	0.999
Nausea & vomiting	6 (12.8)	0 (0.0)	6 (8.0)	0.078
Surgical site infection	3 (6.4)	0 (0.0)	3 (4.0)	0.289
Acute renal failure	1 (2.1)	0 (0.0)	1 (1.3)	0.999
**Readmission [*n*, (%)]**	3 (6.4)	0 (0.0)	3 (4.0)	0.289
**Reoperation [*n*, (%)]**	3 (6.4)	1 (3.6)	4 (5.3)	0.999

Significant values are italics.

*SPSG* single-port sleeve gastrectomy, *RPSG* reduced port sleeve gastrectomy, *CLSG* conventional laparoscopic sleeve gastrectomy.

### Postoperative pain

Comparison between the groups found similar NRS scores for postoperative pain on the operation day (6.4 ± 1.3 vs. 6.4 ± 1.7, *P* = 0.726), postoperative day 1 (4.0 ± 1.5 vs. 4.3 ± 1.5, *P* = 0.330), day 2 (3.4 ± 1.0 vs. 3.5 ± 1.3, *P* = 0.629), and day 3 (2.8 ± 1.1 vs. 2.6 ± 1.1, *P* = 0.245; Table 6).

**Table 6:** Postoperative pain NRS score in the total and propensity score-matched cohorts.

	Total cohort				Matched cohort			
	SPSG + RPSG (*n* = 75)	CLSG (*n* = 73)	Total (*n* = 148)	*P*-value	SPSG + RPSG (*n* = 50)	CLSG (*n* = 50)	Total (*n* = 100)	*P*-value
Day of operation [mean ± SD]	6.4 ± 1.3	6.4 ± 1.7	6.4 ± 1.5	0.726	6.6 ± 1.1	6.6 ± 1.7	6.6 ± 1.4	0.993
Postoperative day 1 [mean ± SD]	4.0 ± 1.5	4.3 ± 1.5	4.2 ± 1.5	0.330	4.0 ± 1.6	4.4 ± 1.6	4.2 ± 1.6	0.256
Postoperative day 2 [mean ± SD]	3.4 ± 1.0	3.5 ± 1.3	3.4 ± 1.1	0.629	3.5 ± 1.1	3.6 ± 1.4	3.5 ± 1.2	0.596
Postoperative day 3 [mean ± SD]	2.8 ± 1.1	2.6 ± 1.1	2.7 ± 1.1	0.245	2.8 ± 1.1	2.6 ± 1.2	2.7 ± 1.1	0.452

*NRS* numeral rating scale, *SPSG* single-port sleeve gastrectomy, *RPSG* reduced port sleeve gastrectomy, *CLSG* conventional laparoscopic sleeve gastrectomy, *SD* standard deviation.

### Resolution of obesity-related comorbidities

The resolution of obesity-related comorbidities was evaluated 12 months after surgery (Table 7). The CLSG group showed higher dyslipidemia remission (17 [37.0%] vs. 28 [63.6%], *P* = 0.018) before matching. After propensity score matching, the groups showed no difference in comorbidity resolution. Of the entire cohort, 13 patients (17.3%) in the SPSG + RPSG group and 24 (32.9%) in the CLSG group developed GERD symptoms de novo after sleeve gastrectomy (*P* = 0.029). After matching, CLSG group patients showed higher rate of newly developed reflux esophagitis on endoscopy after surgery (4 [8.5%] vs. 12 [26.1%], *P* = 0.025). Twelve patients (16.0%) in the SPSG + RPSG group and five (6.8%) in the CLSG group developed hiatal hernia after surgery (P = 0.121). None of the patients required surgical correction.

**Table 7:** Resolution of comorbidities in the total and propensity score-matched cohorts.

	Total cohort				Matched cohort			
	SPSG + RPSG (*n* = 75)	CLSG (*n* = 73)	Total (*n* = 148)	*P*-value	SPSG + RPSG (*n* = 50)	CLSG (*n* = 50)	Total (*n* = 100)	*P*-value
**Hypertension [*n*, (%)]**				0.809				0.687
Remission, complete	10 (25.0)	13 (28.3)	23 (26.7)		10 (41.7)	11 (34.4)	21 (37.5)	
Remission, partial	15 (37.5)	13 (28.3)	28 (32.6)		5 (20.8)	10 (31.3)	15 (26.8)	
Improvement	12 (30.0)	17 (37.0)	29 (33.7)		7 (29.2)	10 (31.3)	17 (30.4)	
Unchanged	3 (7.5)	3 (6.5)	6 (7.0)		2 (8.3)	1 (3.1)	3 (5.4)	
**Diabetes [*n*, (%)]**				0.826				0.859
Remission, complete	15 (68.2)	17 (60.7)	32 (64.0)		7 (58.3)	13 (65.0)	20 (62.5)	
Remission, partial	2 (9.1)	2 (7.1)	4 (8.0)		1 (8.3)	2 (10.0)	3 (9.4)	
Improvement	4 (18.2)	8 (28.6)	12 (24.0)		4 (33.3)	5 (25.0)	9 (28.1)	
Unchanged	1 (4.5)	1 (3.6)	2 (4.0)		0 (0.0)	0 (0.0)	0 (0.0)	
**Dyslipidemia [*n*, (%)]**				*0.018*				0.180
Remission	17 (37.0)	28 (63.6)	45 (50.0)		12 (42.9)	18 (64.3)	30 (53.6)	
Improvement	25 (54.3)	14 (31.8)	39 (43.3)		16 (57.1)	10 (35.7)	26 (46.4)	
Unchanged	4 (8.7)	1 (2.3)	5 (5.6)		0 (0.0)	0 (0.0)	0 (0.0)	
Aggravated	0 (0.0)	1 (2.3)	1 (1.1)		0 (0.0)	0 (0.0)	0 (0.0)	
**GERD [*n*, (%)]**								
Resolution								
Symptoms only	3 (4.0)	1 (1.4)	4 (2.7)	0.620	2 (4.0)	1 (2.0)	3 (3.0)	0.999
Esophagitis on endoscopy	5 (6.7)	1 (1.4)	6 (4.1)	0.209	5 (10.0)	1 (2.0)	6 (6.0)	0.204
De novo diagnosis								
Symptoms only	13 (17.3)	24 (32.9)	37 (25.0)	*0.029*	8 (16.0)	15 (30.0)	23 (23.0)	0.096
Esophagitis on endoscopy	9 (13.4)	16 (24.6)	25 (18.9)	0.101	4 (8.5)	12 (26.1)	16 (17.2)	*0.025*

Significant values are italics.

*SPSG* single-port sleeve gastrectomy, *RPSG* reduced port sleeve gastrectomy, *CLSG* conventional laparoscopic sleeve gastrectomy, *GERD* gastroesophageal reflux disease.

### Operative time, cost, and intraoperative complications

Operation time showed no difference between the SPSG + RPSG group and the CLSG group (117.4 ± 37.3 vs. 122.9 ± 45.8 min, *P* = 0.523). Cost analysis showed that the SPSG + RPSG group and the CLSG group were comparable in cost related to the procedure (3925 ± 2380 vs. 3840 ± 3135 USD, *P* = 0.877). There was one case of intraoperative bleeding from the splenic hilum in the SPSG + RPSG group, which led to multi-port conversion. No other intraoperative complications were found. In both groups, sleeve gastrectomy was successfully performed without conversion to laparotomy.

## Discussion

Obesity is a growing global health concern. Bariatric surgery is the treatment of choice for patients who have failed in making the change through lifestyle interventions and medical therapy^15^. The earliest data on bariatric surgery came from the United States and European countries. Several studies have reported ethnic differences in postoperative weight loss outcomes^16,17^. Although some studies on Asian cohorts found laparoscopic sleeve gastrectomy to be feasible and safe, its effectiveness in Asians remains to be confirmed^17–19^.

After its first introduction in gynecologic and urologic procedures, SILS gained increasing acceptance in bariatric procedures, including sleeve gastrectomy^7,20^. The main advantages of SILS include minimal muscle trauma and thus reduced postoperative pain, shorter hospital stay, and improved cosmetic results^20,21^. Multiple studies have reported that SPSG showed equivalent outcomes in weight loss and postoperative morbidity to the CLSG technique^22^. Nonetheless, whether SPSG increases the risk of postoperative complications including leakage, bleeding, and incisional hernia needs to be further elucidated^23^.

In this study, we found that SPSG and RPSG showed weight loss outcomes similar to CLSG. The groups were similar throughout the follow-up period in weight loss, BMI reduction, %TWL, and %EWL. %EWL after one year was 87.4%, 94.4%, and 75.2% in the SPSG, RPSG, and CLSG groups, respectively. These results were superior to an EWL of approximately 70% reported in recent studies^24,25^. The groups were also similar in complication rates. The most feared complications of sleeve gastrectomy are staple line leakage and bleeding; there was only one case of postoperative bleeding that required surgical revision in the multi-port group. No case of leakage was observed. Subgroup analysis revealed differences in early minor complications between the SPSG and RPSG groups. These were all cases of nausea and vomiting that required medication for symptom control.

Postoperative pain showed similar results in both groups, consistent with previous reports^21^. After propensity score matching, the groups were similar in obesity-related comorbidity resolution rate. DM remission rates were 68.2% and 60.7% in the SPSG + RPSG and CLSG groups, respectively, higher than rates reported in previous studies^26^. De novo development of GERD after sleeve gastrectomy is an important postoperative issue, and contributing factors include shape of the sleeve, extent of injury to the lower esophageal sphincter, and presence of hiatal hernia^27,28^. Severe GERD symptoms are associated with both physical and emotional problems, and both obesity and GERD are responsible for increased rate of adenocarcinoma in the cardia^29^. Therefore, optimal sleeve construction without modification of anatomical anti-reflux mechanisms is critical. In the present study, we found that SPSG + RPSG patients showed a slightly lower tendency to develop GERD de novo. This could be explained by the learning curve effect for optimal sleeve construction. Repeated performance of sleeve gastrectomy could have led to a better sleeve shape with optimal diameter and preservation of the antrum. As early cases were exclusively multi-port, this could explain the slightly higher de novo GERD rate in the CLSG group.

Although SPSG is increasingly applied in obese patients, indications for the procedure are not established. Patient selection for SPSG is crucial since the transumbilical approach can be very difficult in tall patients with deep abdominal cavities^30^. Previous studies suggested that patients with a xipho-umbilical distance of > 15 cm or height of > 170 cm should be advised to undergo CLSG^9,20,31^. Mittermair et al. proposed limiting SPSG to patients with a BMI of < 45 kg/m^2^^32^. Hiatal hernia, GERD, extremely high BMI (> 40 kg/m^2^), and previous upper gastrointestinal surgery were suggested contraindications^9,33^. Our study set the recommended indication for SPSG as female patients with BMI ≤ 42 kg/m^2^ and no history of upper abdominal surgery except for laparoscopic cholecystectomy. With these inclusion criteria, SPSG and RPSG results were similar to those of CLSG. Large-scale prospective studies are needed to further develop a globally accepted standard criteria.

The operating surgeon’s learning curve in SPSG is prolonged because handling the laparoscopic instruments in a confined and restricted space is technically demanding^34^. RPSG resolves the technical challenges of the SPSG technique while maintaining the CLSG principles^9^. A comparison of SPSG and RPSG patients found no difference in weight loss or postoperative morbidity. Therefore, RPSG could be an alternative option in selected cases.

Conversion to CLSG was needed in one case (0.7%) due to intraoperative bleeding from the splenic hilum. The patient recovered without further complications and was discharged on the scheduled date. The reasons for conversion from SPSG to CLSG in previous studies were technical difficulties, including insufficient endostapler length, poor visualization, and intraoperative bleeding^8^. Although SPSG is a safe choice for selected patients, multi-port conversion should always be considered when needed. Extra ports should not be considered a failure of the single-port technique, as adding supplementary trocars during conventional laparoscopic procedures is never considered a failure^22^.

This study has certain limitations. First, we evaluated the short-term outcomes of SPSG, RPSG, and CLSG at 1, 3, 6, and 12 months after surgery. Studies with an extended follow-up period could offer additional information on the weight reduction effect and late-onset complications of the single-port or reduced port approach. Second, improved cosmesis is one of the primary advantages of SILS; however, analysis for scar satisfaction was not included in our study. In addition, the study was limited by its retrospective design. Lastly, there might have been a potential selection bias in the SPSG and RPSG patients, and future studies are needed to further validate standardized criteria for selecting single-port and reduced-port candidates.

SPSG and RPSG in selected Asian patients were as safe, feasible, and effective as CLSG, with comparable postoperative weight loss, morbidity, pain, and resolution of comorbidities. Operative time, cost, and intraoperative complications showed no difference between the groups. The SPSG and RPSG approaches could be an alternative in selected patients. However, multi-port conversion should always be considered when needed during surgical procedures.

## Supplementary Information

Supplementary Tables.

Supplementary Figures.
