# Supplementary material for: Safety and effectiveness of reduced-port laparoscopic sleeve gastrectomy in Asian morbidly obese patients
Source: Sci Rep. 2021 Dec 6;11:23511. doi: 10.1038/s41598-021-02999-1 (PMC8648717; doi:10.1038/s41598-021-02999-1)
Supplement: Supplementary file 1 — Supplementary Tables. [file 41598_2021_2999_MOESM1_ESM.docx]

| **Supplementary Table S1. Baseline characteristics of the single-port and reduced-port laparoscopic sleeve gastrectomy patient subgroups.** | | | | |
| --- | --- | --- | --- | --- |
|  | Single-port (*n* = 47) | Reduced-port (*n* = 28) | Total (*n* = 75) | *P*-value |
| **Sex [*n*, (%)]** |  |  |  | 0.067 |
| **Female** | 35 (74.5) | 26 (92.9) | 61 (81.3) |  |
| **Male** | 12 (25.5) | 2 (7.1) | 14 (18.7) |  |
| **Age [yr, mean ± SD]** | 37.9 ± 11.4 | 38.1 ± 11.6 | 38.0 ± 11.4 | 0.922 |
| **Height [cm, mean ± SD]** | 163.9 ± 8.8 | 162.5 ± 6.5 | 163.4 ± 8.0 | 0.467 |
| **Weight [kg, mean ± SD]** | 103.3 ± 22.9 | 97.6 ± 11.7 | 101.2 ± 19.6 | 0.157 |
| **BMI [kg/m^2^, mean ± SD]** | 38.1 ± 5.8 | 37.0 ± 3.8 | 37.7 ± 5.1 | 0.290 |
| **Comorbidities [*n*, (%)]** |  |  |  |  |
| **Hypertension** |  |  |  | 0.543 |
| **No** | 24 (51.1) | 11 (39.3) | 35 (46.7) |  |
| **Diagnosed** | 18 (38.3) | 12 (42.9) | 30 (40.0) |  |
| **Incidentally found** | 5 (10.6) | 5 (17.9) | 10 (13.3) |  |
| **Diabetes** |  |  |  | 0.318 |
| **No** | 31 (66.0) | 22 (78.6) | 53 (70.7) |  |
| **Diagnosed** | 12 (25.5) | 6 (21.4) | 18 (24.0) |  |
| **Incidentally found** | 4 (8.5) | 0 (0.0) | 4 (5.3) |  |
| **Dyslipidemia** |  |  |  | 0.138 |
| **No** | 22 (46.8) | 7 (25.0) | 29 (38.7) |  |
| **Diagnosed** | 11 (23.4) | 7 (25.0) | 18 (24.0) |  |
| **Incidentally found** | 14 (29.8) | 14 (50.0) | 28 (37.3) |  |
| **NAFLD** | 22 (46.8) | 10 (35.7) | 32 (42.7) | 0.470 |
| **Psychological disorder** | 12 (25.5) | 7 (25.0) | 19 (25.3) | 0.999 |
| **GERD** |  |  |  |  |
| **Symptoms only** | 0 (0.0) | 3 (10.7) | 3 (4.0) | *0.049* |
| **Esophagitis on endoscopy** | 11 (23.9) | 3 (10.7) | 14 (18.9) | 0.160 |
| **OSA** | 8 (17.0) | 7 (25.0) | 15 (20.0) | 0.552 |
| **Lung disease** | 2 (4.3) | 0 (0.0) | 2 (2.7) | 0.526 |

SD, standard deviation; BMI, body mass index; NAFLD, non-alcoholic fatty liver disease; GERD, gastroesophageal reflux disease; OSA, obstructive sleep apnea
