# Supplementary figures and images for: Safety and effectiveness of reduced-port laparoscopic sleeve gastrectomy in Asian morbidly obese patients
Source: Sci Rep. 2021 Dec 6;11:23511. doi: 10.1038/s41598-021-02999-1 (PMC8648717; doi:10.1038/s41598-021-02999-1)

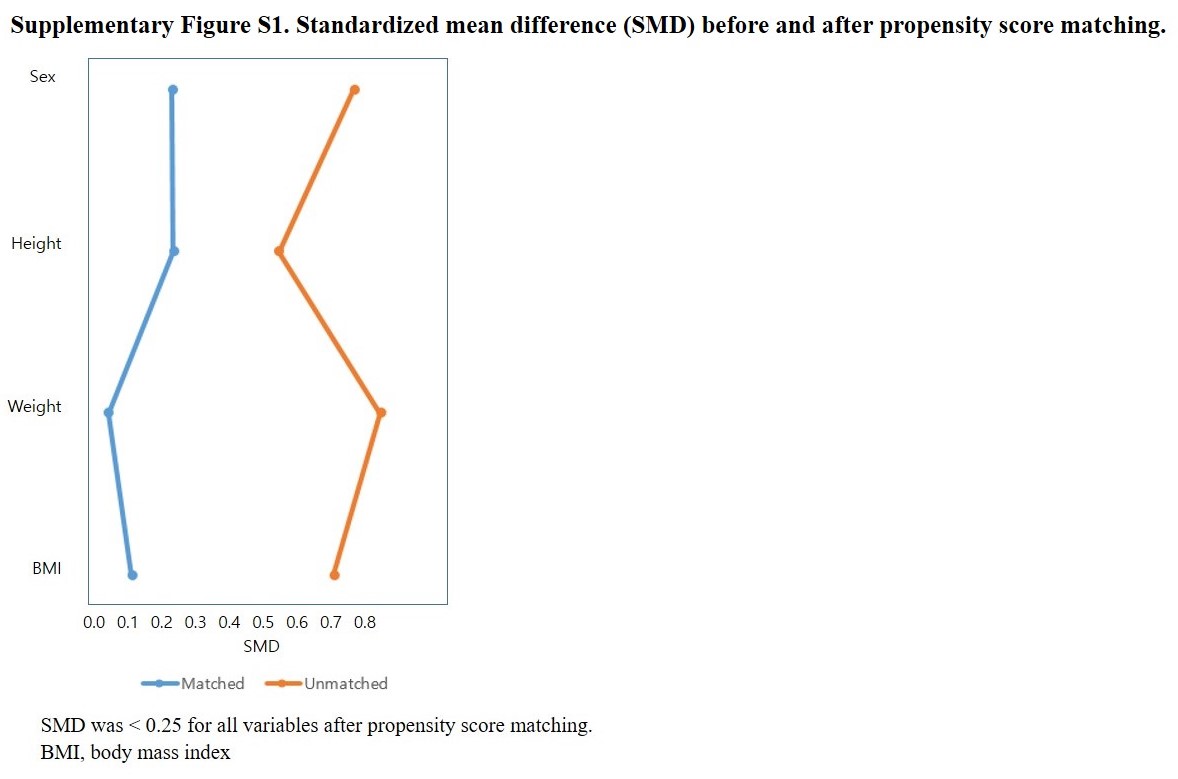

Supplement: Supplementary file 2 — Supplementary Figures. [file 41598_2021_2999_MOESM2_ESM.jpg]
